# The Populations' Resilience Toward the Policymaking Discrepancies in the Pandemic Covid-19 Period

**DOI:** 10.3389/fpubh.2021.733519

**Published:** 2021-10-12

**Authors:** Hatem H. Alsaqqa

**Affiliations:** Ph.D. Health Services Management, Ministry of Health, Gaza, Palestine

**Keywords:** COVID-19, pandemic, policymaking, resilience, crisis, population, behavior

## Abstract

The world is in the midst of a crisis unlike any other in recent memory. COVID-19 is a pandemic that is urgent, global in scope, and has huge consequences. The policy sciences provide insights into unfolding trends, and this article uses the lessons of the literature to better understanding the policymaking shifts and population acceptability of COVID-19. The author attempts to investigate how policymakers' emotions and narratives affect policy decisions and form policymaker-population relationships. The author addresses policymaking processes, transitions, interpretations of policy responses, policy implementation through multilateral topics and evaluating policy progress and failure. Trust is linked to cultural norms, values, and faiths in policy literature, and it is seen as a component of key social and economic policy outcomes. The author ends by identifying understudied facets of policymaking that need to be addressed during pandemics.

## Background

Since the spread of infection is multifactorial, countries that are equipped with a multipronged strategy perform better in managing a pandemic like COVID-19. It is worth noting that policymaking is focused in part on empirical experience, which was problematic in the case of COVID-19. When COVID-19 first appeared in early 2020, little was understood about the virus, its disease dynamics, or its consequences. As a result of the lack of information about COVID-19, evidence may be confused.

Because of the peculiar existence of COVID-19, where facts were not visible, policymakers had the aptitude to misinterpret facts. Wearing masks, banning public meetings, closing academic institutions and public areas, national and international mobility limits, confinement and a variety of other policies were implemented with the aim of flattening the curve. Compelled to respond quickly to the unfolding disease situation, politicians in every country attempted to balance the introduction of containment policies. Lockdowns were generally well-received at the start of the pandemic, with the rhetoric around health, and in reality, tight confinement in COVID-19 was related to increased intention to fulfillment and trust in policymakers (1). With the pandemic spreading and populations becoming more focused and fatigued by the various steps taken originally into consideration by policymakers, the pandemic is becoming more exhausting.

As a result, there has been a lot of debate in every society about whether or not the policymaker's actions were acceptable. Despite the difficulty of enacting such legislation, lawmakers, and public health experts must persuade their citizens to change their behavior and respect future containment steps. As a result, it is important to take into account people's concerns about the pandemic and their views of the repercussions of protection policies so that future policies and response planning are well-informed, and the public can expect a high level of compliance.

As a result, looking at the media will reveal how policymaking has changed over time. The public's growing fatigue with lockdowns aligns with this experience, as opposed to the population's initial positive response to “doing their part” to flatten the curve. The global shock caused by COVID-19 has shown how long-lasting the policy conclusions are. It seems that people are no longer willing to embrace the excuse that problems are too complicated or time-consuming to solve.

Individuals have the opportunity to present their positions in new venues in order to build allies and effect policy change. To summarize, the equilibrium can be punctured by trying to alter policymaking, expanding conflict, and venturing into new policy arenas. The public's acceptance of a program has an effect on its' chances of success (2). Although research has looked into the public acceptability of various frameworks for transmitting policy messages (3) there has been very little nationally representative public research into public acceptance of various health policies.

## The Public Health Policymaking Process in Crisis

Advocates for population health must become more politically astute and pay more attention to health's political indicators. It is important to consider how the social science literature on policy reform can be incorporated into the public health strategy if population health policies are to be effectively developed and implemented (4). The nature of the issue, the scale of the benefits and adverse effects, viability and acceptability, as well as resource and equity implications, all must be carefully considered when making public health decisions.

In the absence of strong scientific evidence, politicians must adhere to basic values that can direct decision-making in order to guide public health best practices. These circumstances provide policymakers with challenges related to decision-making, public knowledge, sense-making, transparency, learning, and change (5), but they also necessitate broad cooperation and teamwork involving numerous individuals and organizations. During complex crises, resolving value differences can lead to public debates and blame games.

The recognition of the need for and application of “sophisticated scientific evidence” is in and of itself, a public health best-practice concept (6). Without accurate knowledge on the virus and its dissemination mechanisms, as well as the efficacy of potential interventions and their (direct and indirect) health and socioeconomic effects, policymakers are tasked with taking steps to protect their populations from the epidemic. However, in a volatile and rapidly evolving world, the relevant evidence is constantly changing, making it difficult to make scientifically sound assumptions about the outcomes of different courses of action (7). This propensity to fixate on a single narrative—or, more broadly, this inability to deal with uncertainty—may lead to the misinterpretation of the condition of COVID-19 outbreak, eventually leading to suboptimal decisions with potentially catastrophic consequences (8).

Moreover, there are uncertainties about the length and termination of policy decisions. Although policymakers are undergoing a wave of policy reforms aimed at addressing urgent social threats, it is unclear which of these changes will be lasting and which will be phased out. This includes concerns about how they will be phased out (in phases or at once) and the political ramifications of reversing decisions that expanded welfare benefits to deal with the immediate crisis (9).

Divergent expert evaluations or varying modeling forecasts have presented policymakers with somewhat different perspectives on possible epidemic scenarios. In the face of such confusion, politicians can try to reconcile the opposing viewpoints, or they can completely embrace one without regard for the possibility that it may greatly distort our fundamental knowledge base (10). Crisis response and management share an immediate interdependence with (1) public policies, including the substance of previously and newly enacted policy decisions, (2) the relationships of people, organizations, coalitions, and channels, and (3) crisis response and management share an immediate interdependence with contextual factors, such as income levels, local interactions, and international decisions.

## Populations Resilience

The COVID-19 pandemic is similar to natural disasters in that both disrupt individual and institutional practices and require society to be resilient. The COVID-19 pandemic is a global health shock, with over three million deaths and over 104 million people sickened by April 18, 2021 (11). The steps taken to monitor and mitigate it have generated a socio-economic shock, endangering people's livelihoods all over the world (12).

In disaster literature, resilience is described as a social system's ability to proactively respond to and recover from disruptions that are interpreted within the system to be beyond the range of usual and expected shocks (13). Increasing adaptability at the maneuver level is the essence of resilience. Literature on resilience looks at how it manifests itself in self-organized systems within societies (14). Simultaneously, resilience is required to work in accordance with current policymaker processes and institutions, to reinforce current practices and most importantly, to adhere to current policy frameworks (15).

Community resilience is described as a dynamic and dialogical process in which communities build, improve, and/or engage their resources in order to cope with shocks and the instability that follows (16). Many countries emerged during COVID-19 in a state of overburdened (health) services, semi-closed economies and a resulting scarcity of services and protective supplies. During this stage of the health crisis, resilience is likely to aid societies in adapting, that is, continuing to work in a “natural” manner as much as possible. In other stages of community resilience, can also assist communities in preparing for a crisis and/or restoring or even transforming after a crisis (17).

However, vulnerability is often thought to be the antithesis of resilience (18). During the COVID-19 pandemic, vulnerabilities refer to the health and socioeconomic effects of the pandemic, in which people are at risk of sickness, loss of normal life, (domestic) abuse, and/or social isolation (19). As the society faces a common challenge, increased population engagement emerges, but it is limited by the loss of community resources. Only vulnerabilities over which communities have control can be addressed, and the emphasis is likely to be on immediate and urgent conflict, prevention, or adaptation (20).

The mechanism of gaining civil acceptance of the lockdown policy in the ongoing COVID-19 emergency is partly dependent on procreating resilience like an all and somehow unavoidable logic (21). In natural disasters and health emergencies, two factors seem to be decisive. On the one hand, when individuals and communities are confronted with a shock, the social capital available within the group will dynamize the available resources of people and communities. The wider governance sense in which the initiative is formulated, on the other hand, may serve as a trigger or a constraint for resilience (22).

## The Perception Behavior of Population Toward Policymaking

People's behavior is also affected by social expectations, such as how others are viewed, as well as moral norms and values. People were inspired to do what was right by social compliance, which allowed them to follow and internalize shared guidelines (23). It is possible to develop more effective and efficient public policy using its ideas and empirical findings, which provide information about how people actually make decisions. Behavioral insights are being implemented into public policy based on this information and complex sets of guidelines are being applied to its development and application in many countries (24).

Individual choices differ depending on situations, location, time, norms and social factors, emotional decisions, cognitive distortions and prejudices, modifying rationale applied principles, and at the same time on how and under what conditions the choice is made (the choice architecture), according to studies conducted on different aspects of human behavior (24). As a result, incorporating morality, solidarity, empathy, and compassion into local campaign messaging to promote pro-social activities, thereby improving population-mindedness and responsibility among individuals, may be successful. Moving forward, healthcare officials and policymakers would need to rely on behavioral and social science evidence to improve connectivity and populations' awareness (23).

Behavioral perspectives gathered from many of the studies suggest that people think in two ways (25). They make relative rather than absolute decisions, acts conceptually in certain cases, and is vulnerable to prejudices and biases; they fail to cope with conflicting information, so they depend on current and readily available data; they make decisions emotionally, spontaneously, and then implement them slowly or not at all; are influenced by other people's actions, relationships with them, and social norms. Understanding the perspective on human behavior, human decisions, and limited reasoning that direct the reactions of public healthcare policy receivers is therefore a critical component of successful policymaking. Human behavior research is guiding policymaking, assisting in the development of innovative ways of action and complementing conventional approaches (i.e., regulations and incentives). Furthermore, behavioral perspectives should be developed to direct potential applications and maintain citizens' trust in government policymaking (24).

## Trust and Transparency

The ability to leverage population cooperation and maintain the behaviors required for controlling the pandemic is dependent on indorsing trust. According to Siegrist and Zingg (26), effective pandemic communication requires high levels of trust based on shared values among participants, as well as trust that future events will unfold as anticipated. Indeed, policymakers should place a premium on accountability, particularly in circumstances where they must act quickly and with minimal consultation for the public good, as is frequently the case in public health emergencies.

However, effectively managing a pandemic necessitates large-scale behavioral adjustments on a personal, organizational, and societal level, which go beyond handwashing, facemasks, and isolation. Furthermore, crises, especially epidemics, create real barriers to efforts to balance individual and collective interests, making it difficult to implement the necessary behavioral strategy to reduce outbreak (27).

A social policy is described as “ongoing strategies for structuring relationships and coordinating behavior to achieve collective goals, ways of exerting control, of persuading people to do things they might not do” (28). The implementation of a policy necessitates the mobilization of resources from wherever they are required to carry out the policy. Sociocultural identity, age, gender and resources access all, affect people's interactions with and responses to public health information and communication.

Policymakers' unwillingness to enact targeted community health communication interventions could jeopardize their aptitudes and the health system's ability to respond to pandemics. Because of inadequate messaging by health officials and other authorities, it has been impossible for the community to differentiate between evidence-based and less objectively accurate knowledge during crisis.

In a public health crisis, transparent communication means revealing what information was used to make public-health decisions, who was consulted, and what possibilities and trade-offs were addressed. Of course, population participation can be difficult in times of crisis, when politicians must make fast, life-saving decisions that may necessitate implementing stringent measures with little to no time for community contribution. These steps can be life-changing in themselves. Also, active and continuing public involvement and collaboration with governments, organizations, and other stakeholders, on the other hand, will help to promote restoration (29).

## The Appropriate Strategies to Engage the Population in Policymaking

Avoiding exposure to a highly transmissible disease or taking care of family, neighbors, and staff may be shared goals; however, the mode of delivery and the framing of vital public health information needs to be sensitive to and tailored toward specific social groups and communities (30). The argue that an effective strategy is a two-way process that involves clear messages, delivered via appropriate platforms, tailored for diverse audiences and shared by trusted people. A diversity of community groups must be included in engagement activities with the implications of emerging digital technologies (31).

The author offers a collection of suggestions for a successful pandemic plan. These suggestions are intended to serve as building blocks for broader strategies that value neighborhood diversity and demonstrate a commitment to community engagement. Many of the guidelines overlap, and there are synergistic and, in some cases, antagonistic relationships between them. The author claims that the guidelines can be used to address other public policy and national issues that cut through social, economic, and health domains. In the aftermath of the COVID-19 pandemic, there are still understudied facets of policy sciences that demand further study. These include, but are not limited to, the following research areas:

- The global response to the pandemic has increased the need for new studies not just on the flood of new policy decisions, but also on the consequences of non-decisions and policy terminations.
- The political response to the pandemic has shifted priorities, resulting in a shift in the focus and intensity of policy disagreements, but the characteristics and long-term consequences of these shifts are unclear.
- Although we know that base principles and other orientations influence policy performance and failure, there are still unanswered questions about how to manage the tradeoffs that exist between them.

## Conclusion

It is critical that policy changes resulting from the COVID-19 crises are wise and long-lasting. Instead of short-sighted and temporary strategies, strategic planning, and foresight should be prioritized (e.g., need for sustained social safety nets). COVID-19 control allows policymakers and their constituents to form mutually supportive relationships based on a common perception of what both sets of actors predict. The ability of policymakers and public health officials to assess how the general public views the efficacy of policymaker responses to COVID-19, as well as particular roles, is critical for recognizing possible roadblocks to disease prevention. This article demonstrates that the policy sciences' strength lies in their ability to provide broad insights into the relationships between policymaking and society.

Nonetheless, given the wide variety of policies implemented by policymakers around the world, it is important to learn not only from new biomedical and epidemiological data, but also from the degree to which these values influenced practice, improved community health, and protected human rights. It is critical to participate early and build greater support and encouragement for policymaking, which is even more applicable to policy reform advocates. Despite the fact that concepts of pluralism make it more difficult to accomplish public policy due to the removal of power, pluralism does offer opportunities to raise problems for discussion and increases the likelihood of changeable reform.

## Author Contributions

The author confirms being the sole contributor of this work and has approved it for publication.

## Conflict of Interest

The author declares that the research was conducted in the absence of any commercial or financial relationships that could be construed as a potential conflict of interest.

## Publisher's Note

All claims expressed in this article are solely those of the authors and do not necessarily represent those of their affiliated organizations, or those of the publisher, the editors and the reviewers. Any product that may be evaluated in this article, or claim that may be made by its manufacturer, is not guaranteed or endorsed by the publisher.
